# On the adaptive significance of group III/IV muscle afferent feedback for the cardiovascular response during sustained locomotion in humans

**DOI:** 10.1113/JP290669

**Published:** 2026-08-03

**Authors:** D. Iannetta, F. G. Laginestra, T. S. Thurston, R. H. Jenkinson, J. Chang, H.-Y. Wan, R. M. Broxterman, J. C. Weavil, M. Amann

**Affiliations:** 1 Department of Anesthesiology, University of Utah, Salt Lake City, Utah, USA; 2 Department of Clinical and Experimental Sciences, University of Brescia, Brescia, Italy; 3 Geriatric Research, Education, and Clinical Center, Salt Lake City VAMC, Salt Lake City, Utah, USA; 4 Department of Internal Medicine, University of Utah, Salt Lake City, Utah, USA

**Keywords:** arterial blood pressure, cardiac output, exercise pressor reflex, leg blood flow, oxygen delivery

## Abstract

While the cardiovascular consequences of group III/IV muscle afferent feedback have been studied during short-duration exercise, its role during sustained (> 5 min) locomotion remains unknown. Thirteen healthy individuals (4f/9m) completed two 20-min cycling bouts at ~50% (moderate) and ~75% (heavy) of ${\dot{V}}_{O_{2}max}$ with intact (CTRL) and pharmacologically attenuated (FENT; lumbar intrathecal fentanyl) group III/IV leg muscle afferent feedback. Cardiac output (CO), mean arterial pressure (MAP), leg blood flow (LBF) and leg vascular conductance (LVC) were quantified. While cardiovascular responses at rest were not different between conditions, afferent blockade reduced CO during the moderate-intensity exercise (*P* = 0.006), with the impact progressively strengthening over time (*P* = 0.027). These effects were absent during the heavy-intensity bout (*P* = 0.741). For moderate-intensity exercise, MAP was consistently ~10% lower (*P* < 0.001) and LVC ~13% higher (*P* = 0.006) during FENT compared to CTRL. During heavy-intensity exercise, MAP was steadily ~14% lower during FENT (*P* < 0.001) and LVC was higher (*P* < 0.001) with progressive increases over time (*P* = 0.019). Afferent blockade had no effect on LBF throughout the moderate-intensity exercise (*P* = 0.407). However, during heavy-intensity exercise, starting from minute 10, there was a between-condition difference, resulting in a significantly higher LBF (~9%) during FENT compared to CTRL (*P* = 0.010). These findings highlight the importance of group III/IV leg muscle afferent feedback for regulating the cardiovascular response to human locomotion but also indicate that its relative contribution varies with the intensity and duration of the exercise.

## Graphical Abstract

Group III/IV leg muscle afferent feedback has previously been recognized as an important determinant of the cardiovascular response to exercise lasting <5 min. Here we demonstrate that the activity of these neurons is crucial in regulating central and peripheral haemodynamic responses during steady state exercise of longer duration. Specifically, when exercise was performed with pharmacologically attenuated feedback from group III/IV leg muscle afferents (via lumbar intrathecal fentanyl), heart rate (HR) was lower throughout the 20-min moderate-intensity exercise (condition effect (*C*)), while stroke volume (SV) and cardiac output (CO) demonstrated a reduction that developed over time (condition × time (*C*×*T*)). During heavy-intensity exercise, while SV and CO were not different between conditions, HR increased more with attenuated feedback. With respect to peripheral haemodynamic responses during moderate-intensity exercise, blocking group III/IV leg muscle afferent feedback reduced arterial blood pressure (MAP) and increased leg vascular conductance (LVC) irrespective of exercise duration and without affecting leg blood flow (LBF). During heavy-intensity exercise, the afferent blockade-induced reduction in MAP remained stable over time, whereas LBF and LVC progressively increased compared to the intact feedback condition. These findings reflect the time-dependent significance of feedback from group III/IV muscle afferents for regulating central and peripheral haemodynamic responses during human locomotion. *Illustration created with* Biorender.com.

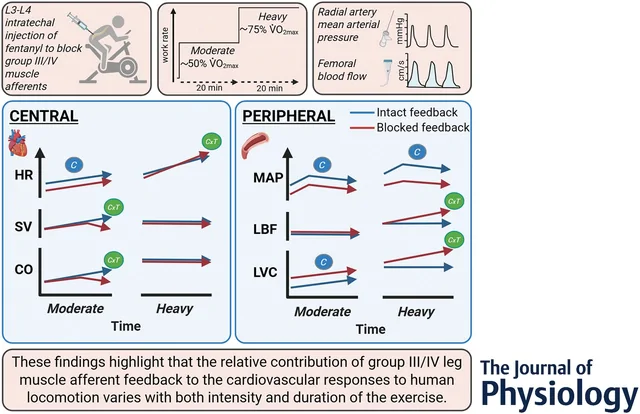

## Introduction

The cardiovascular responses to physical activity are largely determined by neurocirculatory control mechanisms (Fisher, Young and et al. 2015; Wan, Bunsawat and et al. 2023), including central command (Goodwin, McCloskey and et al. 1972), the baroreflex (Bristow, Brown, Cunningham et al. 1971), the chemoreflex (Rutherford & Vatner, 1978), and the exercise pressor reflex (EPR) (Coote, Hilton and et al. 1971; McCloskey & Mitchell, 1972). The EPR is triggered by contraction-induced intramuscular mechanical and metabolic stimuli that increase the discharge frequency of group III/IV muscle afferents. These sensory neurons project to cardiovascular control circuits in the brainstem and, after integration with neural signals from the other control mechanisms, prompt the autonomic nervous system to increase sympathetic and decrease parasympathetic activity. This increases cardiac output (CO) and vascular resistance and thus optimizes the haemodynamic response to exercise (Kaufman & Hayes, 2002; Mitchell, Kaufman and et al. 1983).

Historically, the EPR was conceptualized as a mechanism that monitors the intramuscular environment of exercising skeletal muscle. According to this traditional view, mechanical and metabolic disturbances trigger a group III/IV-mediated ‘error signal’ to the medulla that reflexively adjusts arterial pressure and peripheral perfusion to ensure adequate $O_{2}$ supply to meet the demand of working muscle and vital organs (Mitchell, Kaufman and et al. 1983; Sheriff, Wyss, Rowell et al. 1987). However, more recent animal and human studies challenge this reactive model, suggesting instead that perpetual feedback from group III/IV muscle afferents is continuously engaged in determining optimal cardiovascular responses to exercise (Iannetta, Laginestra, Wray et al. 2025; Kaufman, 2012; Secher & Amann, 2012). This paradigm shift positions the EPR as a ‘proactive’ rather than a purely reactive mechanism.

Human evidence supporting this idea comes from studies employing lumbar intrathecal fentanyl administration to attenuate the spinal transmission of feedback from group III/IV leg muscle afferents. Following this pharmacological intervention, CO and arterial blood pressure are consistently blunted during exercise, while leg vascular conductance (LVC) is increased – especially during light-to-moderate activities characterized by a (quasi) metabolic equilibrium within muscle tissue (Amann, Blain, Proctor et al. 2010; Amann, Venturelli, Ives et al. 2014; Sidhu, Weavil, Venturelli et al. 2015; Thurston, Weavil, Georgescu et al. 2023). Although the exact haemodynamic consequences depend on the intensity and muscle mass engaged in the activity (Thurston, Weavil, Georgescu et al. 2023), the collective findings from these investigations demonstrate that group III/IV muscle afferent feedback serves as a critical determinant of cardiovascular responses across the entire exercise intensity spectrum.

However, the available data – from both animal and human studies – are derived from investigations using relatively short bouts of exercise (≤ 5 min), thus information on the role of group III/IV muscle afferents during longer physical activity is missing. The assumption that feedback from these sensory neurons maintains consistent functional consequences throughout prolonged exercise is problematic and may even be incorrect. Specifically, several competing cardiovascular influences undergo time-dependent changes during prolonged exercise, potentially inducing adjustments in the function of neurocirculatory control mechanisms. For instance, functional sympatholysis may become more pronounced with exercise duration (DeLorey & Clifford, 2022; Thomas & Segal, 2004), intrinsic cardiac inotropic responses may evolve over time (Laginestra, Berg, Nyberg et al. 2023; Middleton, Shave, George et al. 2006), the concentration of vasodilators may change with sustained activity (Gliemann, Vestergaard Hansen et al. 2019), and thermal effects progressively intensify as muscle temperature rises (Pearson, Low, Stöhr et al. 2011; Watanabe, Koch Esteves et al. 2024). Such temporal modifications may result in the relative contribution of group III/IV muscle afferent feedback to the cardiovascular response varying substantially with exercise duration.

To address this gap in our understanding of the EPR, we compared cardiovascular responses to prolonged cycling exercise under control conditions and following pharmacological blockade of group III/IV leg muscle afferent feedback. We hypothesized that group III/IV muscle afferent feedback plays a significant role in regulating the cardiovascular response to exercise, and that their influence on the central and peripheral haemodynamic response is sensitive to both exercise duration and intensity.

## Methods

### Ethical approval

The study was conducted in accordance with the *Declaration of Helsinki* (except for registration in a database) and was approved by the Institutional Review Board of the University of Utah and the Salt Lake City Veterans Affairs Medical Centre (IRB no. 62889). All participants gave written consent to participate in this study.

### Participants

Thirteen recreationally active individuals (age: 21 ± 3 years; height: 176 ± 7 cm; body mass: 70 ± 9 kg; maximal oxygen consumption (${\dot{V}}_{O_{2}max}$): 46 ± 8 ml·kg^−1^ min^−1^; 4 females) participated in this study. Participants were non-smokers, unmedicated and free of overt cardiorespiratory disease. To minimize the effects of hormonal fluctuations, female participants were tested during the early follicular phase of their menstrual cycle (Iannetta, Weavil, Laginestra et al. 2024). All participants signed a consent form prior to their enrolment in this study. Results related to the ventilatory effects of attenuating feedback from group III/IV muscle afferents have previously been published (Iannetta, Weavil, Laginestra et al. 2024).

### Protocol

With each visit separated by ≥48 h, participants completed 4 days of testing, at the same time of the day (±30 min) to perform an incremental test (20 W + 20 W/min) to determine peak work rate (*W*_PEAK_) and ${\dot{V}}_{O_{2}max}$, a familiarization session, and two experimental trials. During the familiarization session, participants completed two consecutive 20-min cycling bouts at ~30% (78 ± 19 W) and ~50% (130 ± 32 W) of *W*_PEAK_ to evoke respectively moderate- and heavy-intensity exercise (Iannetta, Inglis, Mattu et al. 2020). On separate days and in a counterbalanced order, participants completed the same exercise protocol under either of the following two conditions: (1) with intact (control condition; CTRL) and (2) with pharmacologically attenuated (lumbar intrathecal fentanyl) group III/IV leg muscle afferent feedback (FENT). During both experimental sessions, a catheter was inserted into the radial artery to allow for continuous arterial pressure monitoring and blood sampling. To assess potential cephalad migration of fentanyl, participants also performed two consecutive 3 min bouts of arm cycling at 15 W and 30 W immediately before and after the injection. All cycling protocols were completed on a cycle ergometer (Velotron, Racer Mate, Seattle, WA, USA) at a target cadence of 75 rpm. Pulmonary, neuromuscular and other data collected during the experimental visits have recently been published elsewhere for a different experimental question (Iannetta, Weavil, Laginestra et al. 2024).

#### Cardiovascular responses.

Leg blood flow (LBF) was quantified every five minutes throughout the moderate- and heavy-intensity bouts of exercise. Femoral artery blood velocity and diameter were recorded approximately 2–3 cm proximally from the superficial/deep bifurcation using a Doppler ultrasound in duplex mode and equipped with a linear array transducer function (imaging frequency 14 MHz) (Logiq 10) General Electric Medical Systems, Milwaukee, WI, USA). Artery diameter was determined as the wall-to-wall peak diameter coincidental to the peak electrocardiogram R-wave. Four measurements of artery diameter were obtained and ensemble averaged for each time point of interest using a custom-made application. Blood velocity was recorded during the one minute prior to each time point of interest using a Doppler frequency of 5 MHz and a high-pulsed repetition frequency mode (2–25 MHz) with a corrected insonation angle of 60° and the sampling volume maximized and centred according to vessel size and position in real-time. LBF (ml/min) was calculated as: (mean blood velocity) × *π* × (vessel radius)^2^ × 60 s.

Diastolic and systolic blood pressure were continuously recorded using a pressure transducer placed in the radial artery. Mean arterial pressure (MAP) was calculated as the average of the arterial pressure waveform. LVC was calculated as follows: LVC = LBF/MAP. Heart rate (HR) was determined from the R–R interval measured by a 12-lead electrocardiogram (CardioCard; Nasiff Associates, Central Square, NY). Stroke volume (SV) was estimated using the pulse contour method (i.e. ModelFlow) of the arterial pressure tracing (Wesseling, Jansen, Settels et al. 1993). This approach considers the non-linear aortic pressure-area relationship using a three-element model of aortic input impedance (aortic characteristics impedance, arterial compliance, and total peripheral resistance) (Dawson, Shave, Whyte et al. 2007) while assuming constancy of elastance and dimension across repeated measurements (Gratz, Kraidin, Jacobi et al. 1992). CO was calculated by multiplying SV and HR. Systemic vascular conductance (SVC) was calculated as the ratio between CO and MAP. It should be mentioned that the ability of the ModelFlow method to track changes in CO over time, even without a physiological calibration (Azabji Kenfack, Lador et al. 2004; Dawson, Shave, Whyte et al. 2007; Jansen, Schreuder, Mulier et al. 2001), is comparable to established techniques such as cardiac ultrasound and right-heart catheter-based thermodilution (de Wilde, Schreuder et al. 2007; Harms, Wesseling, Pott et al. 1999; Sugawara, Tanabe, Miyachi et al. 2003; van Lieshout, Toska et al. 2003). Thus, although this method is not without limitations regarding the determination of absolute CO, the repeated-measures, within-subject design employed in the current study strengthens confidence in the relative treatment (i.e. afferent blockade) effects, as each participant served as their own control. In addition, pulse contour analysis of the catheter-derived arterial waveforms likely mitigated the impact of any systematic inaccuracies of the method (Azabji Kenfack, Lador et al. 2004; Wesseling, Jansen, Settels et al. 1993).

#### Blood analyses.

During each experimental session, blood from the arterial catheter was drawn at rest and during exercise to measure total haemoglobin content (tHb), haemoglobin $O_{2}$ saturation ($H_{b}O_{2}$) and arterial $O_{2}$ tension $\left(P_{{aO}_{2}}\right)$ (GEM 5000, Instrumentation Laboratories, Bedford, MA, USA). These samples were collected every 5 min throughout each intensity bout. Arterial blood $O_{2}$ content $\left(C_{{aO}_{2}}\right)$ (mL/dL) was calculated as follows: ($\left(1.39\;mL\;O_{2}/g\;Hb\times (tHb)\times \left(H_{b}O_{2}\right)\right)+((0.003\;mL\left.O_{2}/dL\right)\times P_{{aO}_{2}})$.

#### Intrathecal fentanyl and arm cycling.

While seated, participants received an intrathecal injection of fentanyl (0.025 mg) at the L3–L4 vertebral interspace (Amann, Runnels, Morgan et al. 2011b). Importantly, fentanyl could ascend within the cerebrospinal fluid and influence cardiovascular responses by acting directly on medullary opioid receptors (Lalley, 2008). To assess the possibility of cephalad migration – which might confound interpretation of the results – participants performed two 3-min bouts of arm-cranking exercise at 15 W and 30 W using a mechanically braked arm ergometer (Monark-Crescent, Varberg, Sweden). These bouts were conducted prior to and again 10 min after the injection of fentanyl (Amann, Blain, Proctor et al. 2010). A post-injection decrease in minute ventilation (${\dot{V}}_{E}$) greater than two standard deviations (SD) below the pre-injection baseline level was considered indicative of cephalic migration (Thurston, Weavil, Georgescu et al. 2023). In such instances, the participant would be excluded from subsequent data analyses.

### Statistical analyses

Data are presented as mean ± SD. Student’s paired *t* tests were used to determine potential differences at baseline between CTRL and FENT. Mixed-effects linear regression models were employed to account for repeated measures within subjects, with subject as a random effect (Aarts, Verhage, Veenvliet et al. 2014). Robust standard errors were calculated to account for clustering at the subject level. Each outcome was analysed using a three-way interaction model with condition (CTRL *vs*. FENT), exercise intensity (50% *vs*. 75% of ${\dot{V}}_{O_{2}max}$) and time (minutes: 5, 10, 15, 20) as categorical fixed effects. We used post-estimation contrasts to test both marginal effects and interaction effects for: (1) the three-way interaction (condition × intensity × time), (2) the two-way interaction of condition × intensity, (3) the two-way interaction of condition × time at each intensity, and (4) simple effects of condition at each intensity level. *Post hoc* pairwise comparisons were conducted at each time point between conditions within a given intensity, with *P* values adjusted for multiple comparisons using the Hommel procedure (Sankoh, Huque and et al. 1997; Wright, 1992). Statistical significance was set at an *α* level of 0.05. All statistical analyses were performed using Stata (Stata 17, StataCorp LLC, College Station, TX, USA).

## Results

### Arm cycling exercise and metabolic and ventilatory responses during leg cycling

Fentanyl injection had no effect on the ventilatory response to arm cycling exercise in any of the 13 participants (15 W: ~26 L min^−1^; 30 W: ~32 L min^−1^). As published elsewhere (Iannetta, Weavil, Laginestra et al. 2024), ${\dot{V}}_{O_{2}}$ (*P* = 0.118) and ${\dot{V}}_{{CO}_{2}}$ (*P* = 0.119) during exercise were not different between conditions, while ${\dot{V}}_{E}$, ${\dot{V}}_{E}/{\dot{V}}_{O_{2}}$ and ${\dot{V}}_{E}/{\dot{V}}_{{CO}_{2}}$ were all lower during FENT compared to CTRL (*P* < 0.001). ${\dot{V}}_{O_{2}}$ recorded at the end of each intensity corresponded to 52 ± 3% and 77 ± 5% of ${\dot{V}}_{O_{2}max}$.

### Central haemodynamics

**Figure 1.**
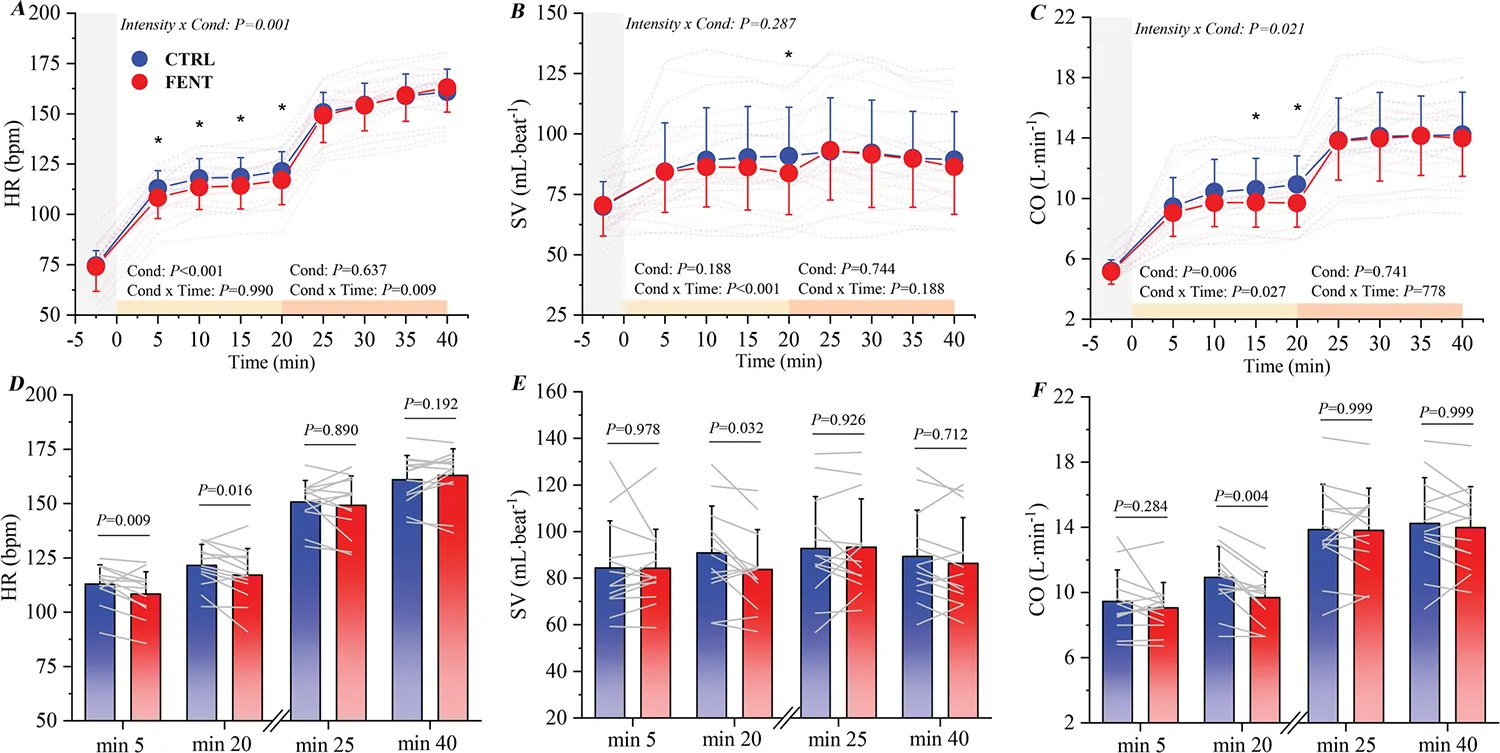
Central haemodynamic responses with intact and attenuated feedback Central haemodynamic responses during constant-load cycling exercise corresponding to ~50% and ~75% of ${\dot{V}}_{O_{2}max}$, performed with intact (CTRL) and attenuated (FENT) group III/IV leg muscle afferent feedback. The upper panels reflect group averages (+SD) and individual data. The grey shaded areas highlight measures at baseline while the yellow (moderate intensity) and orange (heavy intensity) horizontal bars reflect the duration at each exercise intensity. The lower panels illustrate group mean and individual data (+SD) at minute 5, 20, 25 and 40. These time points were selected to represent differences accruing over time. Statistics from the mixed-effects linear regression models are presented for all comparisons. HR, heart rate; SV, stroke volume; CO, cardiac output. *N* = 13 (4 females). ∗Difference between conditions *(P* < 0.05).

HR, SV and CO at rest and during exercise are displayed in Fig. 1 and Table 1. At rest, there was no difference in HR (*P* = 0.660), SV (*P* = 0.837) and CO (*P* = 0.647) between CTRL and FENT.

#### Moderate-intensity exercise.

The HR response was lower during FENT compared to CTRL (*P* < 0.001); however, there was no condition × time interaction (*P* = 0.990), meaning that the decrease in HR was consistent from minute 5 to minute 20 (~4%). While there was no condition effect of fentanyl on SV (*P* = 0.188), a significant condition × time interaction was detected, with SV being lower at minute 20 in FENT compared to CTRL (7 ± 10%; *P* = 0.032). There was a main condition (*P* = 0.006) and a condition × time interaction (*P* = 0.027) effect of fentanyl on CO, with this variable being lower during FENT compared to CTRL at minute 15 (*P* = 0.012) and minute 20 (11 ± 9%; *P* = 0.004).

#### Heavy-intensity exercise.

Fentanyl-blockade had no main condition effect on HR, SV, or CO during exercise corresponding to ~75% of ${\dot{V}}_{O_{2}max}$. However, a condition × time interaction was detected for HR (*P* = 0.009).

### Peripheral haemodynamics, MAP and SVC

**Figure 2.**
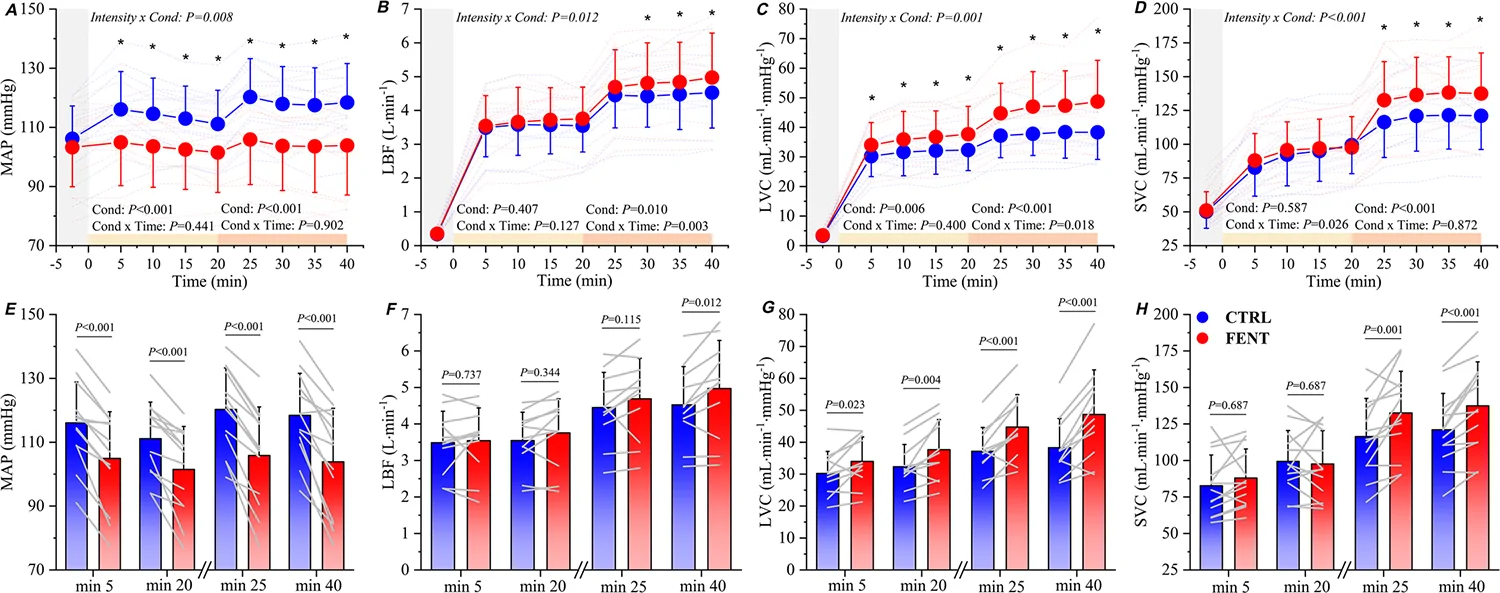
Peripheral haemodynamic responses with intact and attenuated feedback Peripheral haemodynamic responses during constant-load cycling exercise corresponding to ~50% and ~75% of ${\dot{V}}_{O_{2}max}$, performed with intact (CTRL) and attenuated (FENT) group III/IV leg muscle afferent feedback. The upper panels reflect group averages (+SD) and individual data. The grey shaded areas highlight measures at baseline while the yellow (moderate intensity) and orange (heavy intensity) horizontal bars reflect the duration of each exercise intensity. The lower panels illustrate group mean (+SD) and individual data at minute 5, 20, 25, and 40. These time points were selected to represent differences accruing over time. Statistics from the mixed-effects linear regression models are presented for all comparisons. MAP, mean arterial pressure; LBF, leg blood flow; LVC, leg vascular conductance. SVC, systemic vascular conductance. MAP and SVC: *n* = 13 (4 females). LBF and LVC: *n* = 11 (4 females). *Difference between conditions *(P* < 0.05).

Femoral artery diameter at rest was not different between conditions (CTRL = 8.62 ± 1.13 mm; FENT = 8.70 ± 1.19 mm; *P* = 0.067). Furthermore, femoral artery diameter did not change from rest to exercise in CTRL (*P* = 0.410) and FENT (*P* = 0.700) and its overall coefficient of variation across the time points of interest was 1.9 ± 1.3 % in CTRL and 1.6 ± 0.7 % in FENT. MAP, LBF, LVC and SVC at rest and during exercise are displayed in Fig. 2 and Table 1. At rest, there was no difference in MAP (*P* = 0.260), LBF (*P* = 0.743), LVC (*P* = 0.434) and SVC (*P* = 0.630) between CTRL and FENT.

#### Moderate-intensity exercise.

There was a main condition effect of fentanyl on MAP (*P* < 0.001); however, there was no condition × time interaction (*P* = 0.441), meaning that the reduction in MAP was consistent from minute 5 to minute 20 (~10%). Likewise, there was a main effect of fentanyl on LVC (*P* = 0.006) and this was consistent throughout the moderate-intensity bout as the increase in LVC (~16%) was similar across subsequent time points (*P* = 0.400). Fentanyl blockade had no effect on LBF (*P* = 0.407) or SVC (*P* = 0.587) during moderate-intensity exercise.

#### Heavy-intensity exercise.

There was a main condition effect of fentanyl for MAP (*P* < 0.001) with no condition × time interaction (*P* = 0.902). The blockade-induced decrease in MAP was larger during heavy- (~14%) as compared to moderate-intensity (~9%) exercise (*P* < 0.007). There was a main effect of fentanyl on LVC (*P* < 0.001). Furthermore, there was a significant condition × time interaction effect (*P* = 0.018), with the afferent blockade-induced increase in LVC progressively rising over time (minute 5: 21 ± 15%; minute 20: 27 ± 18%). The blockade-induced increase in LVC was overall larger during heavy- as compared to moderate-intensity exercise (*P* = 0.001). Fentanyl had a main condition effect on LBF during the heavy-intensity bout (*P* = 0.010). Moreover, a condition × time interaction was detected (P = 0.003), with LBF at minute 30 (*P* = 0.018), 35 (*P* = 0.035) and 40 (*P* = 0.011) being higher during FENT compared to CTRL. SVC was higher during FENT compared to CTRL (*P* < 0.001), however, there was no condition × time interaction (*P* = 0.693).

## Discussion

This study investigated the cardiovascular relevance of the EPR during prolonged steady-state locomotor exercise by partially blocking signals from group III/IV leg muscle afferents via lumbar intrathecal fentanyl. The data suggest that feedback from these sensory neurons facilitates central haemodynamics during moderate-intensity exercise. While this effect was initially negligible at that intensity, it progressively increased with time. However, group III/IV-mediated afferent feedback had no effect on the central haemodynamic responses during heavy exercise. Furthermore, group III/IV muscle afferent feedback significantly and consistently contributed to the blood pressure response throughout both moderate- and heavy-intensity exercise; the magnitude of this effect was greater at 75% compared to 50% of ${\dot{V}}_{O_{2}max}$. The findings further indicate that group III/IV muscle afferents constrain LVC, with this restriction increasing with both exercise duration and intensity. This led to progressive limitations in LBF at 75% of ${\dot{V}}_{O_{2}max}$, with minimal restrictive effects at moderate intensities or during the initial phase of heavy-intensity exercise. These observations provide additional evidence that group III/IV-mediated feedback optimizes the haemodynamic response to human locomotion. Moreover, the data suggest that the relative contribution of the EPR to the cardiovascular response modulates with exercise intensity and duration, highlighting the varying nature of its influence during sustained physical activity.

### Relative contribution of the EPR to the central haemodynamic response

While generally supporting the previously documented significance of the EPR for modulating the cardiac response to relatively short physical activities, the present study, which was based on long-duration locomotor exercise, provides direct evidence that this influence is both intensity- and duration-dependent. Specifically, although afferent blockade reduced heart rate throughout the entire moderate-intensity bout (Fig. 1A and D), the impact on stroke volume only gradually appeared over time and reached significance at minute 20 of the exercise (Fig. 1B and E). The combination of these changes resulted in an overall reduction in CO, which was significantly lower at minute 15 and 20 of the moderate-intensity bout in FENT compared to CTRL (Fig. 1C and F). During the heavy intensity, afferent blockade had no effect on the cardiac response to exercise (Fig. 1A–F). These findings suggest that the chronotropic and SV effect of the EPR is limited to moderate-intensity exercise, but also highlight that the influence on SV is not initially apparent as it appears over time.

The observed chronotropic effect of the EPR during the moderate-intensity exercise (Fig. 1A and D) aligns with most previous studies based on cycling (Amann, Blain, Proctor et al. 2010) and knee-extension exercise in healthy young and older individuals (Amann, Blain, Proctor et al. 2011a; Amann, Runnels, Morgan et al. 2011b; Sidhu, Weavil, Venturelli et al. 2015). Interestingly, in the only study in which group III/IV leg muscle afferent blockade did not blunt the heart rate response to moderate-intensity exercise (Thurston, Weavil, Georgescu et al. 2023), the normally occurring afferent blockade-induced hypoventilation (Amann, Blain, Proctor et al. 2010, 2011a; Iannetta, Weavil, Laginestra et al. 2024) was prevented by coaching the subjects to raise minute ventilation and match the ventilatory response to that seen during control exercise. It was speculated that the lack of an effect on heart rate might be explained by the extra voluntary ventilatory drive (i.e. higher central command) during the exercise with blocked muscle afferents (Thurston, Weavil, Georgescu et al. 2023). Therefore, while part of the reduction in heart rate during FENT (Fig. 1A and D) may have resulted from the decrease in ventilatory drive associated with the lower minute ventilation (for details, see Iannetta, Weavil, Laginestra et al. (2024), the majority is likely attributable to changes in the activity of the autonomic nervous system. Specifically, by largely eliminating the EPR, afferent blockade compromised the normally occurring decrease in the inhibitory effect of the vagus nerve on the sinoatrial node and the sympathetically mediated activation of *β*-adrenergic receptors (Wan, Bunsawat and et al. 2023) which, in combination, reduced the heart rate response during the moderate-intensity exercise.

Consistent with previous findings using locomotor exercise in healthy humans (Amann, Blain, Proctor et al. 2010, 2011a; Amann, Runnels, Morgan et al. 2011b; Hureau, Weavil, Thurston et al. 2019; Sidhu, Weavil, Venturelli et al. 2014; Sidhu, Weavil, Mangum et al. 2017; Smith, Joyner, Curry et al. 2022), afferent blockade did not affect heart rate during the heavy-intensity bout, despite the significant reduction in minute ventilation. While the exact reasons underlying this lack of effect remain unclear, the predominant role of central command in regulating cardiac sympathetic nerve activity during heavy-intensity exercise likely explains this observation (Nobrega, O’Leary, Silva et al. 2014). This interpretation is supported by studies demonstrating that the influence of central command on the heart rate response during high exercise intensity is sufficiently robust to maintain a normal chronotropic response in the face of an absent EPR (Amann, Proctor, Sebranek et al. 2008; Pawelczyk, Pawelczyk, Warberg et al. 1997).

Afferent blockade evoked a time-dependent effect on SV during the moderate-intensity exercise, but had no impact during heavy-intensity exercise. Specifically, in agreement with earlier findings during locomotor exercise in healthy humans (Smith, Joyner, Curry et al. 2022; Thurston, Weavil, Georgescu et al. 2023), the SV response at minute 5 of the 50% ${\dot{V}}_{O_{2}max}$ bout (i.e. moderate intensity) was similar between FENT and CTRL. However, a between-condition difference developed by minute 20 (Fig. 1B and E). The available data are insufficient to determine the mechanisms underlying this time-dependent, group III/IV-mediated inotropic effect. However, increases in ventricular filling time resulting from afferent blockade-induced reductions in heart rate may have initially compensated for the absent EPR-mediated activation of *β*-adrenergic receptor on ventricular myocardial cells, which normally facilitates ventricular contractility and stroke volume (O’Leary & Augustyniak, 1998). Additionally, although afferent blockade might have decreased right atrial pressure (Sheriff, Augustyniak and et al. 1998), and subsequently impaired ventricular filling and stroke volume, the concurrent increase in filling time could have initially masked this negative impact (O’Leary & Augustyniak, 1998). Taken together, while the increased ventricular filling time in FENT may have adequately compensated for stroke volume reductions caused by both the diminished cardiac sympathetic activation and the compromised Frank-Starling mechanism during the initial phase of the moderate-intensity exercise, this compensatory effect presumably diminished over time, resulting in a progressively lower stroke volume during FENT compared to CTRL. The absence of an effect of afferent blockade on stroke volume during heavy-intensity exercise likely reflects the predominant influence of central command on regulating cardiac sympathetic nerve activity (Nobrega, O’Leary, Silva et al. 2014) and suggests that the relative contribution of the EPR to regulating CO decreases with exercise intensity.

In summary, this set of results suggests that the EPR modulates cardiac function during locomotor exercise in an intensity- and duration-dependent manner. During moderate intensities, the EPR facilitates both heart rate and stroke volume, with the inotropic effect emerging gradually over time. However, during heavy-intensity exercise, central command likely dominates the regulation of the cardiac responses and the EPR’s influence becomes negligible.

### Relative contribution of the EPR to the peripheral haemodynamic response

During moderate-intensity exercise (~50% of ${\dot{V}}_{O_{2}max}$), afferent blockade consistently reduced MAP and increased LVC without affecting SVC, while LBF was not different between CTRL and FENT (Fig. 2). During heavy-intensity exercise (~75% of ${\dot{V}}_{O_{2}max}$), afferent blockade increased SVC, and the reductions in MAP and the increases in LVC were more pronounced compared to the lower intensity exercise. Throughout the heavy-intensity bout, the between-condition differences in both SVC and MAP remained stable, whereas the difference in LVC progressively widened over time. Concurrently, LBF was not different between conditions at minute 5 of the exercise at ~75% of ${\dot{V}}_{O_{2}max}$. However, while it remained consistent throughout CTRL, it steadily increased from minute 5 during FENT, resulting in a significantly greater hyperaemic response at minute 20 of FENT compared to CTRL (Fig. 2B and E).

Conceptually, corroborating earlier animal (Augustyniak, Collins, Ansorge et al. 2001; Eiken & Bjurstedt, 1987; Wyss, Ardell, Scher et al. 1983) and human (Thurston, Weavil, Georgescu et al. 2023) findings, the current study provides evidence suggesting that the primary mechanism by which the EPR regulates the blood pressure response to locomotor exercise depends on the intensity of the activity. During moderate-intensity exercise, afferent blockade-induced reductions in MAP were primarily mediated by heart rate-driven decreases in CO, as SVC was not affected by the attenuated EPR, despite significant, but relatively small, increases in LVC. Conversely, during the heavy-intensity exercise, the blockade-induced decrease in MAP resulted primarily from reduced total peripheral resistance (as evidenced by the increased SVC), while CO was not affected. The observation that afferent blockade had no effect on SVC during moderate-intensity exercise, but consistently (i.e. no change over time) increased SVC during the heavy-intensity bout reflects an intensity-dependent influence of the EPR on restricting blood flow to non-exercising tissues. These observations suggest that the EPR-mediated sympathetic regulation of MAP undergoes a (likely gradual) shift during locomotor exercise: at low intensities, CO serves as the primary driver, while at higher intensities, characterized by a smaller cardiac reserve, systemic vascular resistance becomes dominant. This transition appears to be governed by metabolic demand (i.e. exercise intensity) and available cardiac reserve – rather than temporal factors.

In contrast to stable responses during CTRL, peripheral haemodynamics progressively changed throughout both intensities in FENT (Fig. 2F and G), indicating that the contribution of the EPR to the hyperaemic regulation increases over time during locomotor activity. Notably, although afferent blockade did not affect SVC (Fig. 2D and H), it consistently elevated LVC during moderate-intensity exercise, while LBF remained unchanged. During heavy-intensity exercise, afferent blockade increased LVC early in the bout, with this effect intensifying toward its end. Interestingly, although LBF did not differ between conditions at minute 5, it was significantly higher in FENT compared to CTRL by minute 20. The lack of an early effect of afferent blockade on LBF at both intensities aligns with previous findings, suggesting that sensory feedback does not substantially influence leg perfusion during short-duration locomotor exercise (Thurston, Weavil, Georgescu et al. 2023).

The mechanisms underlying the evolving influence of group III/IV muscle afferent feedback on LVC and LBF during prolonged locomotor exercise remain unclear. However, intramuscular metabolite accumulation and elevated muscle temperature, both of which promote vasodilatation (Clifford & Hellsten, 2004; Pearson, Low, Stöhr et al. 2011) and raised group III/IV afferent activity (Adreani & Kaufman, 1998; Hertel, Howaldt and et al. 1976; Light, Hughen, Zhang et al. 2008) likely contributed to these changes. Specifically, as the rise in muscle temperature was presumably rather small (Saltin & Hermansen, 1966) and the intramuscular metabolic environment mostly unchanged during moderate-intensity exercise (Cannon, Bimson, Hampson et al. 2014), group III/IV-mediated feedback, and thus the EPR, presumably remained constant throughout. The effect of afferent blockade on LVC and LBF was therefore similar at both minute 5 and minute 20. In contrast, intramuscular metabolites and muscle temperature likely increased throughout the heavy-intensity bout (Cannon, Bimson, Hampson et al. 2014; Saltin & Hermansen, 1966), progressively increasing group III/IV muscle afferent feedback. Consequently, pharmacological blockade of these sensory neurons relieved more afferent feedback, and thus more EPR-mediated vasoconstrictor activity, later *versus* earlier in the bout. As vasodilatory signals from accumulating metabolites and rising temperature were thereby less opposed by vasoconstrictor outflow, LVC and LBF significantly increased over time during the high-intensity bout in FENT. These considerations may explain, at least in part, the time-dependent nature of the EPR’s impact on peripheral haemodynamics during high-intensity exercise.

Changes in arterial $O_{2}$ content can directly modulate muscle blood flow to maintain adequate $O_{2}$ delivery during exercise (Hureau, Weavil, Thurston et al. 2019; Joyner & Casey, 2014; Roach, Koskolou, Calbet et al. 1999). Despite a significant effect of fentanyl-blockade on arterial $O_{2}$ content, the differences were likely negligible (Table 1). Moreover, both haemoglobin saturation (data shown in Iannetta, Weavil, Laginestra et al. 2024) and arterial $O_{2}$ content remained stable over time (i.e. from minute 5 to minute 20 at each intensity; Table 1). Therefore, variations in arterial $O_{2}$ content, either between conditions or with exercise duration, are unlikely to have meaningfully contributed to the changes in LVC and LBF induced by afferent blockade.

## Conclusions

While confirming the intensity-dependent influence of group III/IV leg muscle afferent feedback and, by extension, the EPR on both central and peripheral haemodynamic responses during short-duration locomotor exercise, the current study shows that this neurocirculatory control mechanism remains active throughout prolonged exercise, with its impact evolving over time as exercise progresses. Specifically, group III/IV muscle afferent feedback exerts a duration-dependent influence on CO during moderate-intensity exercise and plays a progressively increasing role in modulating vasomotor tone during heavy-intensity exercise. These observations position the influence of the EPR as dynamically changing depending on the varying homeostatic demands of exercise.

## Supplementary Material

Supplementary material

Supporting information

Additional supporting information can be found online in the Supporting Information section at the end of the HTML view of the article. Supporting information files available:

## Figures and Tables

**Table 1.** Cardiovascular responses at minute 5 and minute 20 of constant-load cycling exercise corresponding to ~50% (moderate) and ~75% (heavy) of ${\dot{V}}_{O_{2}max}$

	Moderate		Heavy	
		
Variable	Min 5	Min 20	Min 5	Min 20

Power output (W)	78 ± 19		130 ± 32	
${\dot{V}}_{O_{2}}$ (%)				
*CTRL*	50 ± 2	52 ± 3	74 ± 5	76 ± 6
*FENT*	51 ± 3	53 ± 3	75 ± 7	77 ± 5
HR (bpm)				
*CTRL*	113 ± 9	122 ± 10	151 ±10	161 ± 11
*FENT*	108 ± 10*	117 ± 12*	149 ± 14	163 ± 12
SV (mL·min^−1^)				
*CTRL*	84 ±20	91 ± 20	93 ± 22	89 ± 20
*FENT*	84 ±17	84 ± 17*	93 ± 21	86 ± 20
CO (L·min^−1^)				
*CTRL*	9.4 ± 1.9	10.9 ± 1.9	13.9 ± 2.8	14.2 ± 2.8
*FENT*	9.1 ± 1.6	9.7 ± 1.6*	13.8 ± 2.6	14.0 ± 2.5
MAP (mmHg)				
*CTRL*	116 ± 13	112 ± 11	120 ± 13	118 ± 12
*FENT*	105 ± 15^#^	102 ± 13^#^	106 ± 15^#^	102 ± 16^#^
LBF (ml·min^−1^)				
*CTRL*	3489 ± 861	3547 ± 776	4450 ± 965	4526 ± 1047
*FENT*	3542 ± 897	3755 ± 935	4691 ± 1106	4973 ± 1316*
LVC (mL·min^−1^·mmHg^−1^)				
*CTRL*	30 ± 7	32 ± 7	37 ± 7	38 ± 9
*FENT*	34 ± 8*	38 ± 9*	45 ± 10^#^	49 ± 14^#^
SVC (mL·min^−1^·mmHg^−1^)				
*CTRL*	83 ± 21	99 ± 21	116 ± 26	121 ± 25
*FENT*	88 ± 20	98 ± 23	133 ± 29*	138 ± 26^#^
$H_{b}O_{2}$ (%)				
*CTRL*	97.8 ± 0.6	97.8 ± 0.7	97.4 ± 0.8	97.6 ± 0.6
*FENT*	97.4 ± 1.1	97.2 ± 0.5*	96.6 ± 0.8*	96.6 ± 1.3*
$C_{{aO}_{2}}$ (mL O_2_·dL^−1^)				
*CTRL*	19.1 ± 1.8	18.9 ± 1.7	19.4 ± 1.7	19.6 ± 1.7
*FENT*	19.1 ± 1.9	18.7 ± 1.9	18.8 ± 2.1	19.0 ± 2.1*

* *P* < 0.05 Denotes difference between conditions for the same time point.

# *P* < 0.001 Denotes difference between conditions for the same time point.

*Note*: Data are presented as mean ± SD. Pre-planned comparisons between conditions and time points were made using a paired-sample *t* test.

Abbreviations: $C_{{aO}_{2}}$, arterial $O_{2}$ content; CO, cardiac output; $H_{b}O_{2}$, haemoglobin saturation; HR, heart rate; LBF, leg blood flow; LVC, leg vascular conductance; MAP, mean arterial pressure; SV, stroke volume; SVC, systemic vascular conductance. Statistics from the mixed-effects linear regression models are presented for all comparisons.

## Data Availability

The data of this study are available from the corresponding author upon reasonable request.

## Key points

- While the cardiovascular significance of group III/IV muscle afferent feedback has been investigated during short-duration exercise (≤5 min), its functional relevance during prolonged locomotion is unknown.
- Using lumbar intrathecal fentanyl to attenuate leg muscle afferent feedback during 20-min cycling bouts at ~50% and ~75% ${\dot{V}}_{O_{2}max}$, we found that these afferents consistently facilitate blood pressure, with effects amplifying with intensity.
- They also progressively restrict leg vascular conductance, particularly during heavy-intensity exercise, creating a cumulative constraint on blood flow that emerges over time rather than immediately.
- We also found that group III/IV muscle-afferents augment cardiac output during moderate-intensity exercise, with this effect strengthening over time. However, this facilitation disappears completely during heavy-intensity exercise.
- These findings demonstrate that muscle afferent feedback dynamically modulates haemodynamic responses during sustained locomotion, with cardiovascular contributions varying substantially based on both exercise intensity and duration, a complexity not apparent in short-duration protocols.
