# Supplementary material for: On the adaptive significance of group III/IV muscle afferent feedback for the cardiovascular response during sustained locomotion in humans
Source: J Physiol. Author manuscript; Available in PMC 2026 Sep 17. (PMC13532924; doi:10.1113/JP290669)
Supplement: Supplementary file 1 [file NIHMS2205804-supplement-Supplementary_material.pdf]

The Journal of Physiology

On the adaptive significance of group III/IV muscle afferent feedback for the cardiovascular response during sustained locomotion in humans

Danilo Iannetta, Fabio Giuseppe Laginestra, Taylor S Thurston, Robert H Jenkinson, Jen Chang, Hsuan-Yu Wan, Ryan M. Broxterman, Joshua C Weavil, and Markus Amann
DOI: 10.1113/JP290669

Corresponding author(s): Danilo Iannetta (danilo.iannetta@unibs.it)

The referees have opted to remain anonymous.

Review Timeline:

Submission Date:	30-Nov-2025
Editorial Decision:	21-Jan-2026
Revision Received:	02-Jun-2026
Editorial Decision:	04-Jul-2026
Revision Received:	06-Jul-2026
Accepted:	09-Jul-2026

Senior Editor: Vaughan Macefield

Reviewing Editor: Igor Fernandes

Transaction Report:

(Note: With the exception of the correction of typographical or spelling errors that could be a source of ambiguity, letters and reports are not edited. Depending on transfer agreements, referee reports obtained elsewhere may or may not be included in this compilation. Referee reports are anonymous unless the Referee chooses to sign their reports.)

Re: JP-RP-2025-290669 "On the adaptive significance of group III/IV muscle afferent feedback for the cardiovascular response during sustained locomotion in humans" by Danilo Iannetta, Fabio Giuseppe Laginestra, Taylor S Thurston, Robert H Jenkinson, Jen Chang, Hsuan-Yu Wan, Ryan M. Broxterman, Joshua C Weavil, and Markus Amann

Dear Dr Iannetta,

Thank you for submitting your manuscript to The Journal of Physiology. It has been assessed by a Reviewing Editor and by 1 expert referee and we are pleased to tell you that it is potentially acceptable for publication following satisfactory major revision.

Please advise your co-authors of this decision as soon as possible.

The referee reports are copied at the end of this email.

Please address all the points raised and incorporate all requested revisions or explain in your Response to Referees why a change has not been made. We hope you will find the comments helpful and that you will be able to return your revised manuscript within 2 months. If your article is NOT for a Special Issue, you may have 9 months to revise. If you require an extension, please contact journal staff: jp@physoc.org. Please note that this letter does not constitute a guarantee for acceptance of your revised manuscript.

Your revised manuscript should be submitted online using the link in your Author Tasks: Link Not Available. This link is accessible via your account as Corresponding Author; it is not available to your co-authors. If this presents a problem, please contact journal staff (jp@physoc.org). Image files from the previous version are retained on the system. Please ensure you replace or remove any files that are being revised.

If you do not wish to submit a revised version of your manuscript, you must inform our journal staff (jp@physoc.org) or reply to this email to request withdrawal. Please note that a manuscript must be formally withdrawn from the peer review process at one journal before it may be submitted to another journal.

TRANSPARENT PEER REVIEW POLICY: To improve the transparency of its peer review process, The Journal of Physiology publishes online as supporting information the peer review history of all articles accepted for publication. Readers will have access to decision letters, including Editors' comments and referee reports, for each version of the manuscript, as well as any author responses to peer review comments. Referees can decide whether or not they wish to be named on the peer review history document.

ABSTRACT FIGURES: Authors are expected to use The Journal's premium BioRender account to create/redraw their Abstract Figures. Information on how to access this account is here: https://physoc.onlinelibrary.wiley.com/journal/14697793/biorender-access.

This will enable Authors to create and download high-resolution figures. If authors have used the free BioRender service, they can use the instructions provided in the link above to download a high-resolution version suitable for publication.

The link provided should only be used for the purposes of this submission. Authors will be charged for figures created on this account if they are not related to this manuscript submission.

LANGUAGE EDITING AND SUPPORT FOR PUBLICATION: If you would like help with English language editing, or other article preparation support, Wiley Editing Services offers expert help, including English Language Editing, as well as translation, manuscript formatting, and figure formatting at www.wileyauthors.com/eeo/preparation. You can also find resources for Preparing Your Article for general guidance about writing and preparing your manuscript at www.wileyauthors.com/eeo/prepresources.

RIGOUR AND REPRODUCIBILITY: We recommend authors consult The Journal's Rigour and Reproducibility requirements to ensure that their manuscript is suitable for publication: https://physoc.onlinelibrary.wiley.com/pb-assets/hub-assets/physoc/documents/TJP-Rigour-and-Reproducibility-Requirements.pdf

REVISION CHECKLIST:

Check that your Methods section conforms to journal policy: https://jp.msubmit.net/cgi-bin/main.plex?form_type=display_requirements#methods.

Check that data presented conforms to the statistics policy: https://jp.msubmit.net/cgi-bin/main.plex?form_type=display_requirements#statistics.

Upload a full Response to Referees file. To create your 'Response to Referees': copy all the reports, including any comments from the Senior and Reviewing Editors, into a Microsoft Word, or similar, file and respond to each point, using font or background colour to distinguish comments and responses and upload as the required file type.

Please upload two versions of your manuscript text: one with all relevant changes highlighted and one clean version with no changes tracked. The manuscript file should include all tables and figure legends, but each figure/graph should be uploaded as separate, high-resolution files.

You may also upload:

- 'Potential Cover Art' for consideration as the issue's cover image
- Appropriate Supporting Information (Video, audio or data set: see https://jp.msubmit.net/cgi-bin/main.plex?form_type=display_requirements#supp).

We look forward to receiving your revised submission.

If you have any queries, please reply to this email and we will be pleased to advise.

Yours sincerely,

Vaughan Macefield
Senior Editor
The Journal of Physiology

REQUIRED ITEMS FOR REVISION

- Author photo and profile. First or joint first authors are asked to provide a short biography (no more than 100 words for one author or 150 words in total for joint first authors) and a portrait photograph. These should be uploaded and clearly labelled together in a Word document with the revised version of the manuscript. See Information for Authors for further details.

- Please upload separate high-quality figure files via the submission form.

- Papers must comply with the Statistics Policy: https://jp.msubmit.net/cgi-bin/main.plex?form_type=display_requirements#statistics.

In summary:

- If n {less than or equal to} 30, all data points must be plotted in the figure in a way that reveals their range and distribution. A bar graph with data points overlaid, a box and whisker plot or a violin plot (preferably with data points included) are acceptable formats.

- If n > 30, then the entire raw dataset must be made available either as supporting information, or hosted on a not-for-profit repository, e.g. FigShare, with access details provided in the manuscript.

- 'n' clearly defined (e.g. x cells from y slices in z animals) in the Methods. Authors should be mindful of pseudoreplication.

- All relevant 'n' values must be clearly stated in the main text, figures and tables.

- The most appropriate summary statistic (e.g. mean or median and standard deviation) must be used. Standard Error of the Mean (SEM) alone is not permitted.

- Exact p values must be stated. Authors must not use 'greater than' or 'less than'. Exact p values must be stated to three significant figures even when 'no statistical significance' is claimed.

- Please include an Abstract Figure file and an Abstract Figure legend. An appropriate figure legend, which should not exceed 150 words in length, should be included in the main manuscript file. The Abstract Figure is a piece of artwork designed to give readers an immediate understanding of the research and should summarise the main conclusions. If possible, the image should be easily 'readable' from left to right or top to bottom. It should show the physiological relevance of the manuscript so readers can assess the importance and content of its findings. Abstract Figures should not merely recapitulate other figures in the manuscript. Please try to keep the diagram as simple as possible and without superfluous

information that may distract from the main conclusion(s). Abstract Figures must be provided by authors no later than the revised manuscript stage and should be uploaded as a separate file during online submission labelled as File Type 'Abstract Figure'. Please also ensure that you include the figure legend in the main article file. All Abstract Figures should be created using BioRender. Authors should use The Journal's premium BioRender account to export high-resolution images. Details on how to use and access the premium account are included as part of this email.

- Please ensure that all figures and tables have a title and legend, and that they have been cited within the main article text.

EDITOR COMMENTS

Reviewing Editor:

Thank you for submitting your work to The Journal of Physiology. Your manuscript was evaluated by an expert reviewer who raised several major concerns that require careful consideration. I agree with the reviewer's assessment and share these concerns regarding key methodological and conceptual aspects of the study. In particular, the reliability of the peripheral blood flow measurements during heavy cycling exercise, the use of Modelflow-derived stroke volume without calibration or supporting reliability data, and the interpretation of the physiological mechanisms underlying the reported responses represent substantial issues that directly affect the central conclusions of the work. These concerns need to be thoroughly addressed before the manuscript can be considered further.

Senior Editor:

Thank you for submitting your manuscript to The Journal of Physiology. It has been assessed by an external reviewer and a reviewing editor, both experts in the field. As you will see, both appreciate the difficl]ulty in undertaking these studies but raise concerns about the methodology. In comments to me, clinical vascular sonographers wonder about the accuracy and reliability of leg blood flow measures using duplex ultrasound. Nevertheless, I would like to give you the opportunity to fully revise your manuscript to allay these concerns. I look forward to receiving your revised submission in due course.

REFEREE COMMENTS

Referee #2:

This paper tested the effect of time and intensity on the relative contribution of the exercise pressor reflex (EPR) to central and peripheral haemodynamic responses to exercise. As stated by the author's, this is additional data not published in Iannetta et al. 2024 (in JPhys).

Generally, I think this paper is very well written, succinct and easy to follow. The research question is important and the experimental design addresses the experimental question. I have two major methodological concerns and additional comments below.

1. It seems incredibly difficult to acquire continuous common femoral artery blood flow during heavy exercise using the methods described. Duplex involves simultaneous blood velocity and diameter measurement, which is difficult to acquire during movement, especially upright cycling. I would like the authors to provide raw .avi files and analyzed diameter and velocity data for appraisal. All peripheral data hinges upon accurate and reliable blood flow measures.

2. Stroke volume via Modelflow is also an area of concern (even from direct arterial waveforms). I am aware it has been validated in several scenarios (and invalidated in others), but it often requires some form of calibration with a more direct method for between-day testing. This is not discussed, nor is reliability data provided. Again, all central haemodynamic data requires accurate and reliable stroke volume measures.

Additional general comment:

1. Could the decrease increase in leg blood flow during heavy exercise FENT simply compensate for decreased CaO_2? Based off these data and previously published work from Iannetta (2024), it looks like Leg O_2 delivery might be unchanged. This is important because it suggests intact group III/IV activity restrains blood flow during prolonged heavy exercise. In the discussion, the author's state, "group III/IV-mediated feedback optimizes the hemodynamic response to human locomotion."... lower leg blood flow in CTRL appears suboptimal. It appears in CNTRL that LBF is stable while in FENT it slightly creeps up, indicating that the increase in LVC is greater than the decrease in MAP on FENT. To me this indicates a vasoactive response to sustain leg O_2 delivery when CaO_2 is decreased and O_2 extraction is challenged.

Introduction:

How is the EPR 'proactive' when the evidence supporting this claim is intrathecal fentanyl (suppresses feedback). The author's don't pose a convincing case that this is a proactive mechanism. I suggest they include additional evidence that refutes general idea that the EPR is a classic feedback mechanism with mechanical and metabolic muscle stimuli.

The hypothesis is vague, lacks direction and is untestable.

Methods:

I don't understand how linearity in LBF was assessed across the 2 workloads. With 2 datapoints the R^2 should be 1.0.

Non-continuous diameter measures are not ideal. Changes in artery diameter can occur throughout progressive exercise.

Stats are clear and appropriate given the experimental model.

Results:

Some dubious outcomes based off the figures, for e.g.:

- Cond x Time for HR during heavy ex

- Cond x Time for SVC during moderate ex

- Cond x Time for LVC during heavy ex

Nevertheless, I think the model is appropriate and may be a superior statistical approach.

Discussion:

Looking at the Saltin paper, it appears as though muscle temperature creeps up during mild and moderate intensity exercise. I don't know if it has been clearly shown that muscle temperature remains at stable during mild- to modertate- intensity

exercise; this would most likely be effected by environmental conditions and convective air flow.

END OF COMMENTS

Reviewing Editor:

Thank you for submitting your work to The Journal of Physiology. Your manuscript was evaluated by an expert reviewer who raised several major concerns that require careful consideration. I agree with the reviewer's assessment and share these concerns regarding key methodological and conceptual aspects of the study. In particular, the reliability of the peripheral blood flow measurements during heavy cycling exercise, the use of Modelflow-derived stroke volume without calibration or supporting reliability data, and the interpretation of the physiological mechanisms underlying the reported responses represent substantial issues that directly affect the central conclusions of the work. These concerns need to be thoroughly addressed before the manuscript can be considered further.

We thank you for this summary and the opportunity to address the concerns raised by the reviewing editor and the external reviewer. We have now responded to all comments in detail and resubmitted an overall improved version of our manuscript.

Senior Editor:

Thank you for submitting your manuscript to The Journal of Physiology. It has been assessed by an external reviewer and a reviewing editor, both experts in the field. As you will see, both appreciate the difficl]ulty in undertaking these studies but raise concerns about the methodology. In comments to me, clinical vascular sonographers wonder about the accuracy and reliability of leg blood flow measures using duplex ultrasound. Nevertheless, I would like to give you the opportunity to fully revise your manuscript to allay these concerns. I look forward to receiving your revised submission in due course.

We thank you for evaluating our manuscript and for the opportunity to address the concerns raised by the reviewer. We appreciate both your and the reviewer's comments regarding blood flow measurements obtained using duplex ultrasound and have made substantial efforts to provide additional information and clarification related to these measurements. We have now addressed all comments and are confident that our responses, together with the revisions made to the manuscript, satisfactorily address the reviewer's concerns.

Referee #2:

This paper tested the effect of time and intensity on the relative contribution of the exercise pressor reflex (EPR) to central and peripheral haemodynamic responses to exercise. As stated by the author's, this is additional data not published in Iannetta et al. 2024 (in JPhys).

Generally, I think this paper is very well written, succinct and easy to follow. The research question is important and the experimental design addresses the experimental question. I have two major methodological concerns and additional comments below.

We want to thank the reviewer for the friendly comment and for carefully evaluating the methodologies employed in this study. We fully appreciate your concerns and comments and are pleased that they have helped improve our manuscript. Please find our point-by-point responses to your comments below.

1. It seems incredibly difficult to acquire continuous common femoral artery blood flow during heavy exercise using the methods described. Duplex involves simultaneous blood velocity and diameter measurement, which is difficult to acquire during movement, especially upright cycling. I would like

the authors to provide raw .avi files and analyzed diameter and velocity data for appraisal. All peripheral data hinges upon accurate and reliable blood flow measures.

Obtaining accurate measures of blood flow during cycling exercise using ultrasonography is indeed technically challenging, and we acknowledge that considerable practice went into developing and mastering the required technical skills. We have previously published studies using this approach (Hureau et al., JAP, 2019; Thurston et al., J Physiol, 2023) and have since further refined our expertise in assessing femoral blood flow during exercise. As we do during the preliminary work for each study that includes blood flow measures via Doppler ultrasound, we have previously demonstrated and published a linear increase in blood flow with progressive increases in workload (Hureau et al., JAP, 2019). Moreover, in this previous study, we observed slight reductions in femoral blood flow during exposure to hyperoxia (FiO_2 = 1.00). Both responses are consistent with the expected physiological behavior of femoral blood flow during exercise and hyperoxia, supporting the sensitivity of this technique to hemodynamic perturbations and its ability to detect subtle differences between experimental conditions.

Regardless, we appreciate your concern and acknowledge that one potential source of uncertainty is whether femoral artery diameter changes from rest to exercise, as well as during prolonged exercise. In the current study, as in our previous work, we assumed that femoral artery diameter remains stable from rest to exercise and therefore used resting diameters for all exercise blood flow calculations. Prompted by your concerns, we re-analyzed arterial diameters at all time points in every participant (rest, and minutes 5, 10, 15, and 20 of each exercise intensity). At each time point, 4 independent diameter measurements were obtained and averaged. A summary of the group mean data is presented in the figure below [the dashed line represents the average diameter at rest (Time 0) projected over the course of the entire protocol].

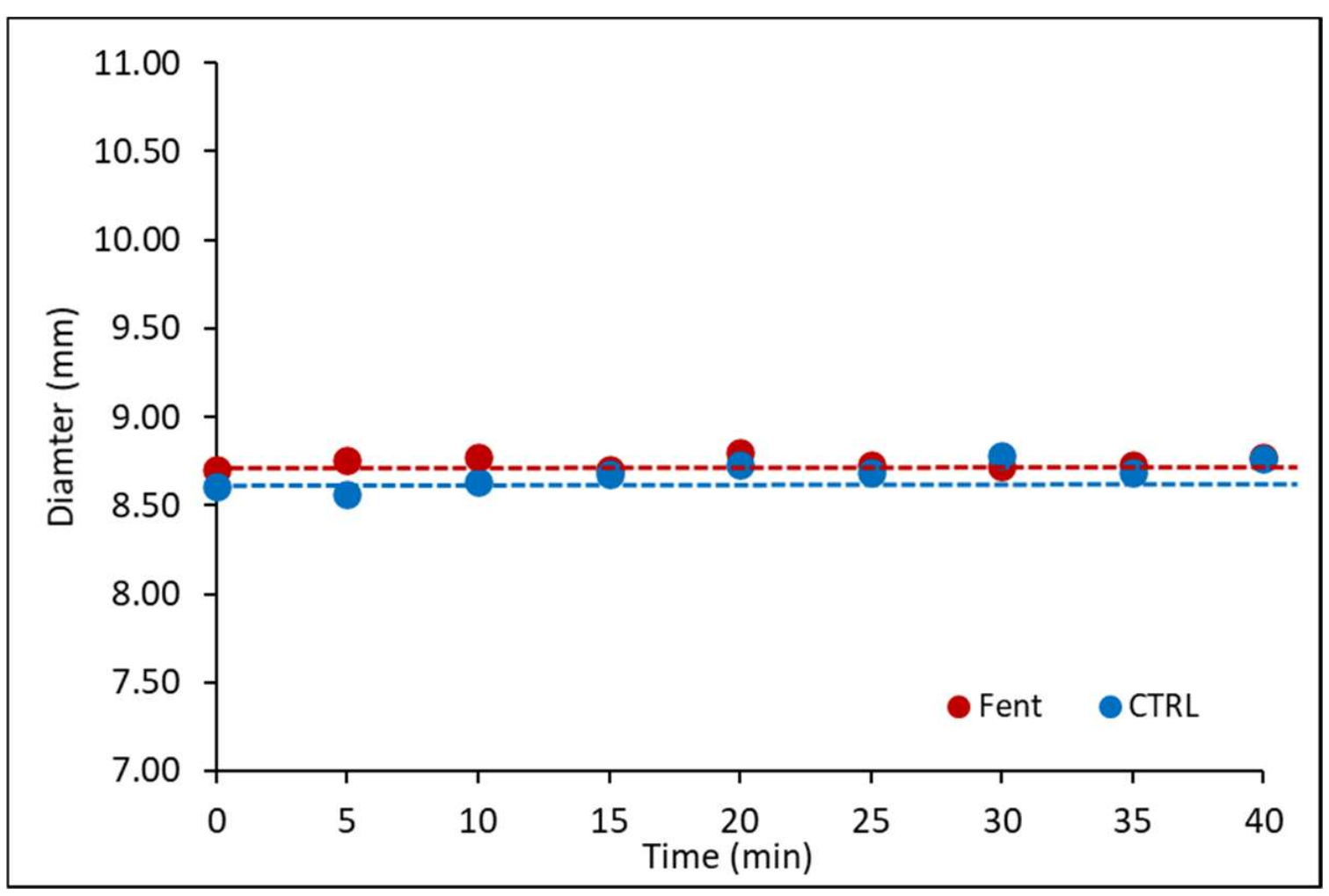

First, the average coefficient of variation across the 4 diameter measurements obtained at each time point was 1.9 % in CTRL and 1.8 % in FENT. Second, after averaging the 4 diameter measurements at each time point, we compared them at rest with each of the diameters during exercise and found no change from rest to exercise in either CTRL (P = 0.409) or FENT (P = 0.700). Third, the average coefficient of variation in artery diameter across all time points corresponded to 0.02 mm in both CTRL and FENT. To put the impact of this variability in context, we applied it to the average femoral artery diameter measured in CTRL (8.6 mm) and FENT (8.7 mm) and found that it corresponded to a 0.5–1.5% change in blood flow, depending on exercise intensity. Finally, we compared the

diameters during FENT exercise with the diameters during CTRL exercise and found them to be not different ($P = 0.420$).

Nonetheless, despite the negligible variability in artery diameter across the time points of interest, we re-calculated all blood flow measurements using the individual artery diameters determined at each time point, rather than applying resting diameters to all exercise measurements. We revised the manuscript accordingly by updating the Methods (Lines 124-125) and Results sections, as well as all relevant figures, tables, and statistical analyses. Although some numerical values changed slightly, the overall findings and conclusions were unaffected.

Finally, to further address the reviewer's concerns, we now provide representative snapshots of diameter recordings from a representative participant at the relevant time points. In addition, we have uploaded two separate videos showing 30-second recordings at each time point of interest for two representative participants. Please note that, due to file size limitations, the videos had to be compressed, resulting in some reduction in image quality. Nevertheless, the quality of the recordings can still be adequately appreciated from the uploaded snapshots.

2. Stroke volume via Modelflow is also an area of concern (even from direct arterial waveforms). I am aware it has been validated in several scenarios (and invalidated in others), but it often requires some form of calibration with a more direct method for between-day testing. This is not discussed, nor is reliability data provided. Again, all central haemodynamic data requires accurate and reliable stroke volume measures.

We appreciate this comment and agree with the reviewer that the results obtained using the ModelFlow approach to derive cardiac output should be interpreted with caution. We are fully aware of the limitations of this method and appreciate that the underlying assumptions may be influenced by several factors. We acknowledge that ModelFlow may have limited accuracy in quantifying absolute cardiac output (L/min) across populations. However, we contend that it reliably captures within-subject changes in response to exercise or pharmacological interventions. Because ModelFlow was applied directly to arterial waveforms and our study employed a repeated-measures, within-subject design, concerns regarding relative changes (evoked by afferent blockade) are minimized. Importantly, this approach also mitigates potential bias in estimating intensity- and fentanyl-induced changes (Δ) in cardiac output within individual participants.

Regarding the absolute accuracy of the estimates produced by ModelFlow, although some studies have questioned its agreement with "gold-standard" methods for cardiac output assessment, several investigations have shown that ModelFlow is comparable, if not superior, to thermodilution and Doppler ultrasound in tracking changes in cardiac output under a variety of conditions, including rest, exercise, and following blood withdrawal (Harms et al., 1999; Sugawara et al., 2003; van Lieshout et al., 2003; de Wilde et al., 2007).

The lack of external calibration in the current study represents a potential limitation. However, prior studies have demonstrated that ModelFlow-derived cardiac output is highly reproducible and capable of reliably tracking changes over time (Wesseling et al., 1993; Azabji Kenfack et al., 2004). These findings suggest that external calibration may not be necessary when the primary focus is on temporal or within-subject changes (Jansen et al., 2001; Azabji Kenfack et al., 2004; Dawson et al., 2007).

That being said, we appreciate the reviewer's concern and, to address this point, we have added a paragraph to the Methods section discussing the potential sources of variability associated with the ModelFlow approach. (Lines 140-149).

Additional general comment:

Could the decrease increase in leg blood flow during heavy exercise FENT simply compensate for decreased CaO2? Based off these data and previously published work from Iannetta (2024), it looks like Leg O2 delivery might be unchanged. This is important because it suggests intact group III/IV activity restrains blood flow during prolonged heavy exercise. In the discussion, the author's state, "group III/IV-mediated feedback optimizes the hemodynamic response to human locomotion."... lower leg blood flow in CTRL appears suboptimal. It appears in CNTRL that LBF is stable while in FENT it slightly creeps up, indicating that the increase in LVC is greater than the decrease in MAP on FENT. To me this indicates a vasoactive response to sustain leg O2 delivery when CaO2 is decreased and O2 extraction is challenged.

Agreed, while reductions in CaO_2 can, in principle, elicit an increase in blood flow, we do not believe that this mechanism explains the progressive increase in blood flow observed from minute 5 to minute 20 during FENT. Specifically, CaO_2 was reduced to a similar extent at minute 5 during FENT compared with CTRL. Despite this reduction, blood flow did not differ significantly between FENT and CTRL at that time point. This observation suggests that the subsequent progressive increase in blood flow during the heavy-intensity exercise bout was not driven solely by the lower CaO_2. Instead, as discussed in our manuscript, we propose that the increase in blood flow is more likely related to a time-dependent change in the contribution of the EPR to the overall vasomotor response.

Introduction:

How is the EPR 'proactive' when the evidence supporting this claim is intrathecal fentanyl (suppresses feedback). The authors don't pose a convincing case that this is a proactive mechanism. I suggest they include additional evidence that refutes general idea that the EPR is a classic feedback mechanism with mechanical and metabolic muscle stimuli.

We appreciate the reviewer's comment but would prefer to keep this admittedly somewhat provocative statement in the manuscript. The following considerations support our postulate. Current evidence suggests that the EPR does not merely elicit a reactive response to perturbations in cardiovascular homeostasis, such as those evoked during the transition from a lower to a higher intensity. Rather, the EPR appears to retain functional relevance even after a metabolic steady state has been established. This perspective challenges the conventional view that the EPR is engaged only during disturbances in skeletal muscle homeostasis. If the latter was the case, we would expect rather unstable cardiovascular responses, meaning, presumably small deviations from the steady state in MAP, conductance, flow, etc (or breathing) would be noticeable, reflecting the trigger re-engaging the EPR to correct this deviation. This is, however, not the case, the responses remain perfectly smooth throughout the entire trial. This could be interpreted that group III/IV afferents are perpetually and continuously active during exercise (which is indeed the case, based on previous Kaufman's studies in animals) and not just when thresholds are breached or disturbances detected. This means cardiovascular adjustments are happening ahead of, or alongside, demand rather than in response to a disturbance. In other words, rather than waiting for an O_2 mismatch to develop (which would alter intramuscular metabolic milieu, i.e., the trigger of group III/IV afferent firing), the EPR appears to be continuously optimizing cardiovascular output, essentially anticipating and fine-tuning the circulatory response in real time rather than correcting deficits after the fact. The phrase "determining optimal cardiovascular responses" is key, it implies the EPR plays an active, governing role in shaping the response, not a corrective one.

The hypothesis is vague, lacks direction and is untestable.

We appreciate this comment and acknowledge that the hypothesis, as stated, is broad. However, we respectfully maintain that the current hypothesis is appropriate given the range of critical variables examined. Formulating a directional hypothesis in this context is not straightforward, as the numerous factors determining the cardiovascular responses would be expected to have directionally different changes. While variable-specific hypotheses (e.g., CO, HR, SV, MAP, LVC, LBF, etc) could be offered, we believe this approach would fragment the narrative, burden the reader unnecessarily, and unduly lengthen the Introduction. We hope the reviewer will appreciate these considerations.

Methods:

I don't understand how linearity in LBF was assessed across the 2 workloads. With 2 datapoints the R2 should be 1.0.

Thanks for this comment. This statement was not pertinent and therefore removed.

Non-continuous diameter measures are not ideal. Changes in artery diameter can occur throughout progressive exercise.

Agreed. We have therefore reanalyzed the data and quantified artery diameter at each time point at rest and during exercise (Lines 124-125; Lines 208-210). Please see our response to one of your previous comments related to the influence of vessel diameter on the quantification of blood flow.

Stats are clear and appropriate given the experimental model.

Thank you.

Results:

Some dubious outcomes based off the figures, for e.g.:

- Cond x Time for HR during heavy ex

- Cond x Time for SVC during moderate ex

- Cond x Time for LVC during heavy ex

Nevertheless, I think the model is appropriate and may be a superior statistical approach.

Thank you for carefully reviewing the accuracy of our graphical results. We have re-examined the statistical analyses and can confirm that all reported results are correct.

Discussion:

Looking at the Saltin paper, it appears as though muscle temperature creeps up during mild and moderate intensity exercise. I don't know if it has been clearly shown that muscle temperature remains at stable during mild- to modertate- intensity exercise; this would most likely be effected by environmental conditions and convective air flow.

Agreed, this is a good point, thank you for pointing this out. We have revised this section to more precisely address the potential for a rise in muscle temperature even during the low intensity exercise (Lines 357-360). Based on the Saltin paper, although muscle temperature appears to be creeping up during the exercise, the reported changes were minimal (<0.5 C).

Dear Dr Iannetta,

Re: JP-RP-2026-290669R1 "On the adaptive significance of group III/IV muscle afferent feedback for the cardiovascular response during sustained locomotion in humans" by Danilo Iannetta, Fabio Giuseppe Laginestra, Taylor S Thurston, Robert H Jenkinson, Jen Chang, Hsuan-Yu Wan, Ryan M. Broxterman, Joshua C Weavil, and Markus Amann

Thank you for submitting your manuscript to The Journal of Physiology. It has been assessed by a Reviewing Editor and by 1 expert referee and we are pleased to tell you that it is acceptable for publication following satisfactory revision.

Please share this decision with your co-authors as soon as possible.

The referee reports are copied at the end of this email.

Please address all the points raised and incorporate all requested revisions or explain in your Response to Referees why a change has not been made. We hope you will find the comments helpful and that you will be able to return your revised manuscript within 4 weeks. If you require longer than this, please contact journal staff: jp@physoc.org.

Your revised manuscript should be submitted online using the link in your Author Tasks Link Not Available. This link is accessible via your account as Corresponding Author; it is not available to your co-authors. If this presents a problem, please contact journal staff (jp@physoc.org). Image files from the previous version are retained on the system. Please ensure you replace or remove any files that are being revised.

If you do not wish to submit a revised version of your manuscript, you must inform our journal staff (jp@physoc.org) or reply to this email to request withdrawal. Please note that a manuscript must be formally withdrawn from the peer review process at one journal before it may be submitted to another journal.

TRANSPARENT PEER REVIEW POLICY: To improve the transparency of its peer review process The Journal of Physiology publishes online, as supporting information, the peer review history of all articles accepted for publication. Readers will have access to decision letters, including Editors' comments and referee reports, for each version of the manuscript, as well as any author responses to peer review comments. Referees can decide whether or not they wish to be named on the peer review history document.

ABSTRACT FIGURES: Authors are expected to use The Journal's premium BioRender account to create/redraw their Abstract Figures. Information on how to access this account is here:
https://physoc.onlinelibrary.wiley.com/journal/14697793/biorender-access.

This will enable Authors to create and download high-resolution figures. If authors have used the free BioRender service, they can use the instructions provided in the link above to download a high-resolution version suitable for publication.

The link provided should only be used for the purposes of this submission. Authors will be charged for figures created on this account if they are not related to this manuscript submission.

LANGUAGE EDITING AND SUPPORT FOR PUBLICATION: If you would like help with English language editing, or other article preparation support, Wiley Editing Services offers expert help, including English Language Editing, as well as translation, manuscript formatting, and figure formatting at www.wileyauthors.com/eeo/preparation. You can also find resources for Preparing Your Article for general guidance about writing and preparing your manuscript at www.wileyauthors.com/eeo/prepresources.

RIGOUR AND REPRODUCIBILITY: We recommend authors consult The Journal's Rigour and Reproducibility requirements to ensure that their manuscript is suitable for publication: https://physoc.onlinelibrary.wiley.com/pb-assets/hub-assets/physoc/documents/TJP-Rigour-and-Reproducibility-Requirements.pdf

REVISION CHECKLIST:

Check that your Methods section conforms to journal policy: https://jp.msubmit.net/cgi-bin/main.plex?form_type=display_requirements#methods

Check that data presented conforms to the statistics policy: https://jp.msubmit.net/cgi-bin/main.plex?form_type=display_requirements#statistics

Upload a full Response to Referees file. To create your 'Response to Referees' copy all the reports, including any comments from the Senior and Reviewing Editors, into a Microsoft Word, or similar, file and respond to each point, using font or background colour to distinguish comments and responses and upload as the required file type.

Please upload two versions of your manuscript text: one with all relevant changes highlighted and one clean version with no changes tracked. The manuscript file should include all tables and figure legends, but each figure/graph should be uploaded

as separate, high-resolution files. The journal is now integrated with Wiley's Image Checking service. For further details, see: https://www.wiley.com/en-us/network/publishing/research-publishing/trending-stories/upholding-image-integrity-wileys-image-screening-service

You may also upload:

- 'Potential Cover Art' for consideration as the issue's cover image
- Appropriate Supporting Information (Video, audio or data set: see https://jp.msubmit.net/cgi-bin/main.plex?form_type=display_requirements#supp).

We look forward to receiving your revised submission.

If you have any queries, please reply to this email and we will be pleased to advise.

Yours sincerely,

Vaughan Macefield
Senior Editor
The Journal of Physiology

REQUIRED ITEMS

- Authors are required to fully disclose the use of any Generative AI (GAI) tools used in the preparation of their manuscript. This disclosure should also be included as a statement alongside the Additional Information and Data Availability statements towards the end of the article under the heading 'Generative AI Statement', and include the name(s), model and version of AI technology used, and exact details on the purpose for, and methods of, its use.

If no AI tool has been used, this should also be specifically stated within the manuscript.

EDITOR COMMENTS

Reviewing Editor:

Congratulations on your revised submission. I agree with the reviewer that the additional analyses, methodological clarifications, and reliability assessments substantially strengthened the manuscript and addressed the major concerns raised during the initial review. The manuscript is considered provisionally accepted pending implementation of a few minor revisions, including incorporation of the reliability/COV data into the manuscript, clarification of velocity averaging procedures, correction of the statement regarding SVC, and consideration of the reviewer's comments regarding muscle temperature.

Senior Editor:

Thank you for submitting your revised manuscript, and detailed responses to the comments, to The Journal of Physiology. AS you will see, Reviewer 1 is largely satisfied with your amendments but, as elaborated by the Reviewing Editor, we would like you to address the small remaining issues before we can accept your paper for publication. I look forward to receiving your revised manuscript in due course.

REFEREE COMMENTS

Referee #2:

The authors have done a considerable amount of re-analysis of ultrasound files and provided reliability data in the response to referee document.

Overall, I think the additional analysis conducted by the author's for the femoral artery ultrasound is satisfactory. There is error in every form of measurement and unlike some techniques, e.g., thermodilution, it is very easy to identify methodological issues with ultrasound because it is visually analyzed. I appreciate the author's including raw files into this resubmission as it makes it obvious that continuous diameter measurement, as is common practice with vascular

assessment ultrasound, e.g. flow-mediated dilation, this would be inaccurate in the current setting. Pending there are no changes in arterial diameter, as is possibility the case here, snapshots of maximum diameter is appropriate. Also, although conduit artery circumference contributes to total blood flow due to it being proportional the the 4th power (as per Poiseuille's law; in most physiological settings, conduit artery blood flow is largely driven by changes in blood velocity because there is so much more capacity to change relative to artery circumference. All this said, I think the authors should present the COV data in the results or methods section to provide the reader confidence in their technique. The authors should also state how long velocity was recorded and averaged, e.g. 1 minute, 15 seconds.

The concerns with Modelflow for stroke volume are still present but it appears there are no additional metrics of stroke volume or cardiac output in this study. This represents a limitation and the author's have identified this in the Methods section. I agree with the author's that ModelFlow has been validated in several settings (which are cited), it's ability to detect subtle differences between placebo and experimental conditions will always be in question. In this study with a small sample size, these differences are subtle and so accurate and reliable tools are essential. I know this is well appreciated by the authors.

Minor points:

The speculation that muscle temperature remains <0.5C from baseline based off the Saltin paper is likely not true (it is 1-2C with light cycling below lactate threshold and up to 5-6C with intense exercise). Unfortunately, I do not have a good published reference to support this claim, besides this MSc thesis that is publicly available. (https://hdl.handle.net/10523/49488). I can appreciate with with the given published evidence, the author's may not wish to modify this section in the discussion.

Line 228 - "SVC was lower during FENT compared to CTRL (P<0.001)..."

-This is incorrect.

END OF COMMENTS

EDITOR COMMENTS

Reviewing Editor:

Congratulations on your revised submission. I agree with the reviewer that the additional analyses, methodological clarifications, and reliability assessments substantially strengthened the manuscript and addressed the major concerns raised during the initial review. The manuscript is considered provisionally accepted pending implementation of a few minor revisions, including incorporation of the reliability/COV data into the manuscript, clarification of velocity averaging procedures, correction of the statement regarding SVC, and consideration of the reviewer's comments regarding muscle temperature.

We want to thank you for the positive feedback regarding the changes we made to our initial submission. We have further amended the manuscript based on the additional reviewer's comments.

Senior Editor:

Thank you for submitting your revised manuscript, and detailed responses to the comments, to The Journal of Physiology. As you will see, Reviewer 1 is largely satisfied with your amendments but, as elaborated by the Reviewing Editor, we would like you to address the small remaining issues before we can accept your paper for publication. I look forward to receiving your revised manuscript in due course.

Thank you for the opportunity to revise our manuscript. We are confident to have successfully addressed all reviewer's comments.

Referee #2:

The authors have done a considerable amount of re-analysis of ultrasound files and provided reliability data in the response to referee document.

Overall, I think the additional analysis conducted by the author's for the femoral artery ultrasound is satisfactory. There is error in every form of measurement and unlike some techniques, e.g., thermodilution, it is very easy to identify methodological issues with ultrasound because it is visually analyzed. I appreciate the author's including raw files into this resubmission as it makes it obvious that continuous diameter measurement, as is common practice with vascular assessment ultrasound, e.g. flow-mediated dilation, this would be inaccurate in the current setting. Pending there are no changes in arterial diameter, as is possibility the case here, snapshots of maximum diameter is appropriate. Also, although conduit artery circumference contributes to total blood flow due to it being proportional the the 4th power (as per Poiseuille's law; in most physiological settings, conduit artery blood flow is largely driven by changes in blood velocity because there is so much more capacity to change relative to artery circumference. All this said, I think the authors should present the COV data in the results or methods section to provide the reader confidence in their technique. The authors should also state how long velocity was recorded and averaged, e.g. 1 minute, 15 seconds.

Thank you for evaluating positively our responses which certainly helped us strengthening our manuscript. Based on the additional requests, we added the coefficient of variations of the diameter recordings (Lines 213-214) and reported the length of the blood flow measurements (Lines 128-129).

The concerns with Modelflow for stroke volume are still present but it appears there are no additional metrics of stroke volume or cardiac output in this study. This represents a limitation and the author's have identified this in the Methods section. I agree with the author's that ModelFlow has been validated in several settings (which are cited), it's ability to detect subtle differences between placebo and experimental conditions will always be in question. In this study with a small sample size, these differences are subtle and so accurate and reliable tools are essential. I know this is well appreciated by the authors.

Agreed. Yes, we very well appreciate these issues and, as mentioned by the reviewer, we have acknowledged the limitations of the ModelFlow method.

Minor points:

The speculation that muscle temperature remains <0.5C from baseline based off the Saltin paper is likely not true (it is 1-2C with light cycling below lactate threshold and up to 5-6C with intense exercise). Unfortunately, I do not have a good published reference to support this claim, besides this MSc thesis that is publicly available.(https://hdl.handle.net/10523/49488). I can appreciate with with the given published evidence, the author's may not wish to modify this section in the discussion.

We appreciate the reviewer's comment. Although the reviewer indicated that the original wording was acceptable, we have removed the "<0.5C" reference to further soften the sentence (Line 360).

Line 228 - "SVC was lower during FENT compared to CTRL (P<0.001)..." -This is incorrect.

Thank you for spotting this typo! Changed. (Line 233).

Dear Dr Iannetta,

Re: JP-RP-2026-290669R2 "On the adaptive significance of group III/IV muscle afferent feedback for the cardiovascular response during sustained locomotion in humans" by Danilo Iannetta, Fabio Giuseppe Laginestra, Taylor S Thurston, Robert H Jenkinson, Jen Chang, Hsuan-Yu Wan, Ryan M. Broxterman, Joshua C Weavil, and Markus Amann

We are pleased to tell you that your paper has been accepted for publication in The Journal of Physiology.

The last Word (or similar) version of the manuscript provided will be used by the Production Editor to prepare your proof. When this is ready you will receive an email containing a link to Wiley's Online Proofing System. The proof should be thoroughly checked and corrected as promptly as possible.

Authors should note that it is too late at this point to offer corrections prior to proofreading. Major corrections at proof stage, such as changes to figures, will be referred to the Editors for approval before they can be incorporated. Only minor changes, such as to style and consistency, should be made at proof stage. Changes that need to be made after proof stage will usually require a formal correction notice.

All queries at proof stage should be sent to: TJP@wiley.com.

Following copyediting and typesetting the accepted version will be published online.

Yours sincerely,

Vaughan Macefield
Senior Editor
The Journal of Physiology

IMPORTANT POINTS TO NOTE FOLLOWING ACCEPTANCE OF YOUR PAPER:

• IMPORTANT NOTICE ABOUT OPEN ACCESS: To assist authors whose funding agencies mandate immediate public access to published research findings, The Journal of Physiology allows authors to pay an Open Access (OA) fee to have their papers made freely available immediately on publication.

The Corresponding Author will receive an email from Wiley with details on how to register or log in to Wiley Authors where you will be able to place an order.

• You can check if your funder or institution has a Wiley Open Access Account here:
https://authors.wiley.com/author-resources/Journal-Authors/open-access/author-compliance-tool.html

MEMBER DISCOUNT: Corresponding authors who are Members of the Physiological Society are eligible for a 20% discount on the cost of the Article Processing Charge (APC). To claim this discount, the Corresponding Author will need to log in to the Physiological Society Membership portal and retrieve the discount code (https://www.physoc.org/journals-and-media/journals/journal-discount-for-members/). This code should then be quoted in the billing section of Wiley Authors when selecting the open access option.

• TRANSPARENT PEER REVIEW POLICY: To improve the transparency of its peer review process, The Journal of Physiology publishes online as supporting information the peer review history of all articles accepted for publication. Readers will have access to decision letters, including Editors' comments and referee reports, for each version of the manuscript as well as any author responses to peer review comments. Referees can decide whether or not they wish to be named on the peer review history document.

• You can help your research get the attention it deserves! Check out Wiley's free Promotion Guide for best-practice recommendations for promoting your work at: www.wileyauthors.com/eeo/guide. You can learn more about Wiley Editing Services which offers professional video, design, and writing services to create shareable video abstracts, infographics, conference posters, lay summaries, and research news stories for your research at: www.wileyauthors.com/eeo/promotion.

• If you would like to receive our 'Research Roundup', a monthly newsletter highlighting the cutting-edge research published in The Physiological Society's family of journals (The Journal of Physiology, Experimental Physiology, Physiological Reports, The Journal of Nutritional Physiology and The Journal of Precision Medicine: Health and Disease), please click this link, fill in your name and email address and select 'Research Roundup':
https://www.physoc.org/journals-and-media/membernews

EDITOR COMMENTS

Reviewing Editor:

Congratulations on this excellent contribution to the field!

Senior Editor:

Thank you for attending to this minor comment. I am pleased to report that your manuscript is now considered acceptable for publication in The Journal of Physiology.
